# Tales from the photovoice clinic: Menopause experiences of Black and Chinese women in the United Kingdom

**DOI:** 10.1177/20533691251346293

**Published:** 2025-05-28

**Authors:** Susan Waigwa, Paula Briggs, Susan Pickard, Elham Amini, Jane Wilkinson

**Affiliations:** 1 Department of Sociology, Social Policy and Criminology, 4591 University of Liverpool, Liverpool, UK; 2 Department of Sociology, Social Policy and Criminology, Liverpool Women’s Hospital, Liverpool, UK

**Keywords:** Menopause, ethnic minority, women’s health, photovoice, black women, chinese women

## Abstract

**Background:**

Menopause is one of many critical life course points that may require intervention in relation to lifestyle changes, health and wellbeing. It is therefore important that menopausal women are supported especially considering possible health inequalities and inclusion issues. While there is increasing recognition for such support, little is known about the experiences of ethnic minority women living in the United Kingdom, who are thought to be less engaged and less likely to seek support, but those who try to seek support experience dissatisfaction.

**Methods:**

This study explored menopause experiences of Black and Chinese heritage women aged over 40, living in the Northwest, England. Photovoice methodology was utilised including a four-phase process: introductory meeting with participants, taking of photos, selection of photos and discussions about the photos. Ten women participated and provided photos that conveyed their experiences with menopause. Photographs and transcripts from discussions were then analysed thematically.

**Results:**

Nine overarching themes were identified across the data: Menopause and management of symptoms including self-care, diet management, exercise, community and menopause and the life course including religion and beliefs, work, family and ageing. These themes captured the challenges and expectation of interacting with healthcare providers, alternative methods of managing menopause, significance of community and focus on the life course.

## Introduction

There is a need to understand menopause transition within a life course context and to appreciate women’s individual biographical histories.^1^ Within the life course, menopause constitutes one of the critical life course points, providing an opportunity to promote lifestyle changes to support health and wellbeing in midlife and post-menopausal women. There is an increasing recognition of the importance of supporting menopausal women not only in the United Kingdom, but globally. The 2030 Agenda for Sustainable Development^2^ is based on the principle of advancing equity and the inclusion of different groups by emphasising the issue of ageing and monitoring gender and health inequalities, to ensure that disadvantaged populations are not excluded from society. However, very little is known about the experience of menopause transition and post menopause in women from diverse ethnic communities living in the United Kingdom.^3^ Less still is known about their perceptions of, and interaction with, healthcare providers around the menopause. The scant previous research in this field suggests that certain ethnic communities are much less likely to interact with healthcare practitioners and that those who do are more likely to express problems and dissatisfactions with their experience.

One of the reasons for the disconnect can be traced back to the evolution of menopause which in Western biomedicine is perceived as a ‘cross-culturally unified’ experience.^4^ Anthropologists have noted that the notion of menopause is not shared or experienced in other cultures in the same way. The concept of ‘local biologies’ reflect the very different social and physical conditions of women’s lives from one society to another.^5^

Aware of these ideologies, our study recognises that menopause contains both a biological/hormonal element and a cultural element.^6^ This perspective preceded our extensive exploration of menopause experiences of women from the Black and Chinese heritage communities living in the Northwest of England. No previous studies had conducted research on these groups using in-depth interviews and creative methods (photovoice), the latter of which this paper presents.

## Methods

### Study design

This photovoice study was conducted between July 2024 and January 2025. Photovoice was originally developed by Wang and Burris^7^ as an action research tool to be used within health promotion research. It is a participatory action research method that uses participant-led photography. This method can give participants (especially silent/marginalised participants) a degree of ownership over research.

### Study setting and selection of participants

This study was conducted in the Northwest of England, which is an ideal site as it is home to Britain’s oldest Black community, established in the eighteenth century and also the oldest Chinese community traceable back to the nineteenth century. Both communities are dynamic, with multiple generations. Ten women (six Black and four Chinese women) aged over 40 were purposefully selected based on their previous participation in in-depth interviews and willingness to continue to participate in the photovoice study.

### Phases of the photovoice process

Phase one involved an introductory meeting held online. The researchers described photo rules, caption and ethical considerations.

Phase two involved participants taking photos (over a two-week period) in relation to (a) menopause; (b) midlife or (c) looking ahead/ageing.

Phase three included selecting at least two of the best photos, giving them headings/captions and sending them via email or phone to the researcher.

Phase four included discussions where seven women attended the in-person workshops (one for Black women and one for Chinese women). The remaining three women provided their discussions individually. Women discussed the titles they chose for each photo, the theme/themes they chose and the reasons for taking the photos. The workshops were recorded.

### The photovoice study process

**Figure 1.**
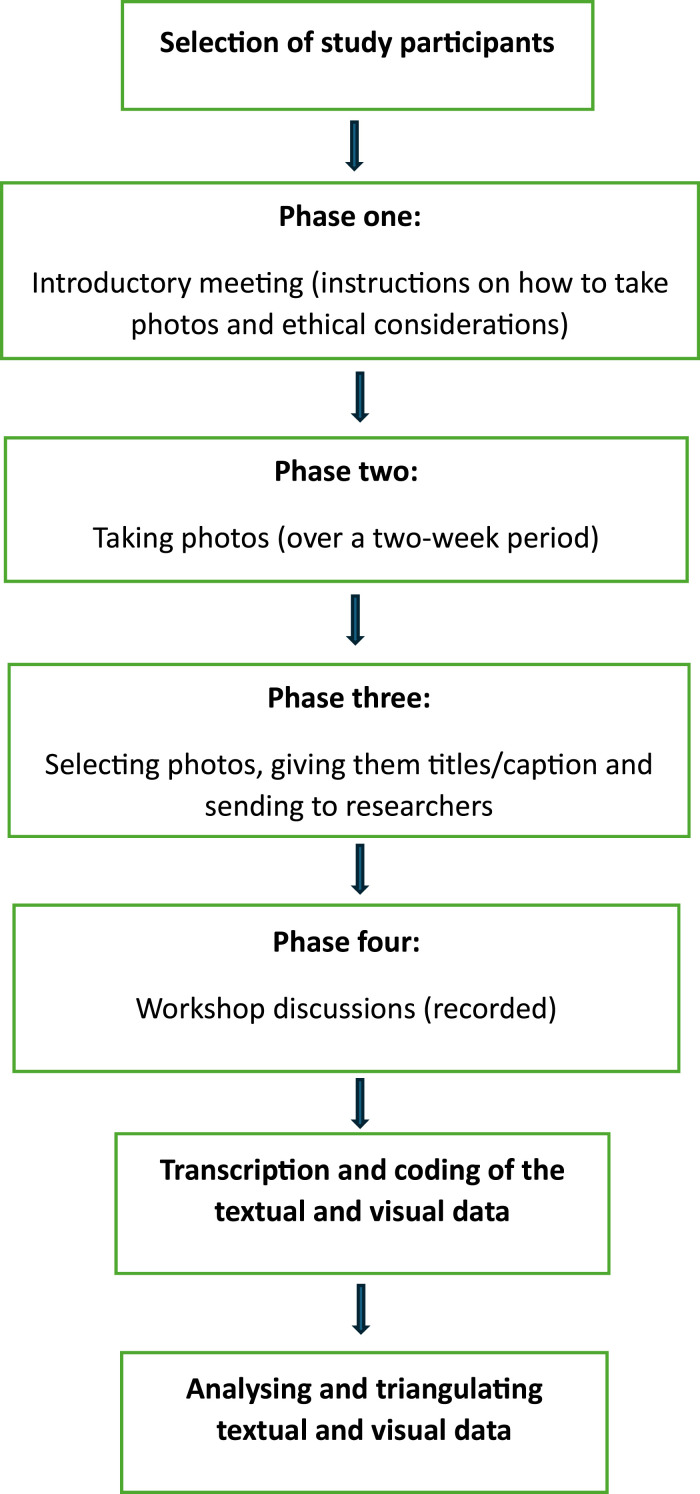
Photovoice study process.

The flowchart (Figure 1) shows the process followed in this photovoice study.

### Ethical consideration

This study was approved by the University of Liverpool ethics committee (Ref number 12920). Written consent to participate and share photos was obtained from each of the participants before commencement of the study.

## Results

Through transcripts, textual and visual data provided by the participants, we identified nine overarching themes unique to the demographics of our participants that depicted their diverse perspectives, experiences and management of menopause, presented here with quotes citing pseudonyms, age and ethnicity origin/heritage:

• Menopause and management of symptoms
  ◦ Symptoms
  ◦ Self-care
  ◦ Diet management
  ◦ Exercise
  ◦ Community
• **Menopause and the life course**
  ◦ Religion and beliefs
  ◦ Work
  ◦ Family
  ◦ Ageing

### Menopause and management of symptoms

Both the Black and Chinese women participants in this photovoice study acknowledged menopause as a natural process that occurs at some point in all women who had menses, although with different symptoms affecting each individual.

**Figure 2.**
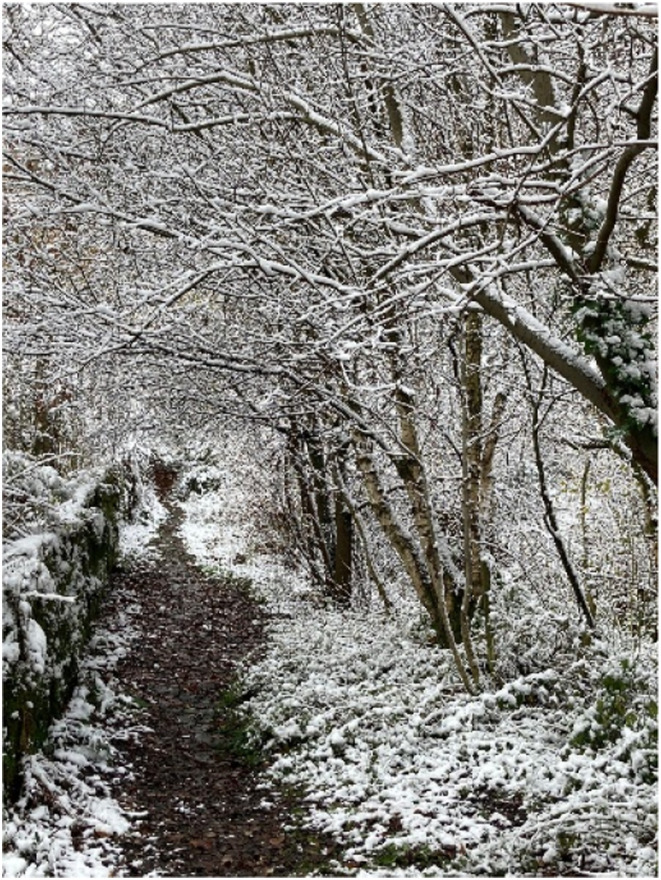
Winter photo presented by Serena.

Some of the women described symptoms that affected them during the menopause transition, which resulted in isolation. Serena (55, Black African origin) indicated that she had been having episodes of heightened anxiety, and she provided a winter photo (Figure 2):

She said: ‘*Sometimes I feel disconnected from the world. I'm hibernating*’.

**Figure 3.**
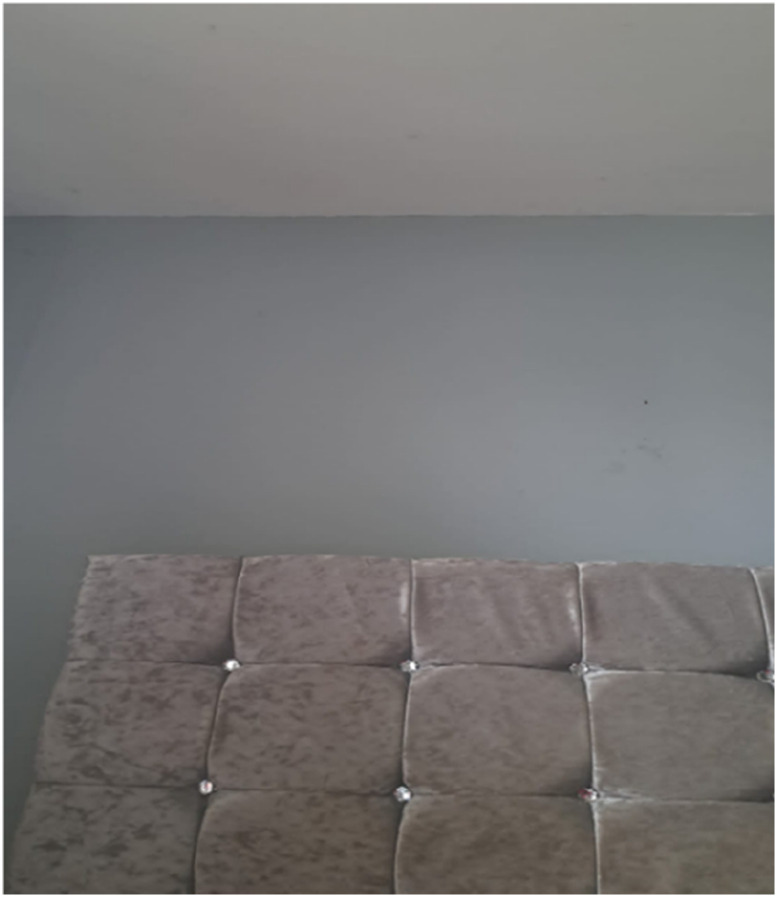
Bed-board photo presented by Anka.

On a similar note, Anka (50, Black Caribbean origin), who also indicated that she was struggling with depression, provided a bed-board photo (Figure 3):

She said: ‘*There is time I never want to leave this room (her bedroom) or socially*’.

Because of such devastating symptoms, some women therefore require help, including medication to cope with the symptoms.

**Figure 4.**
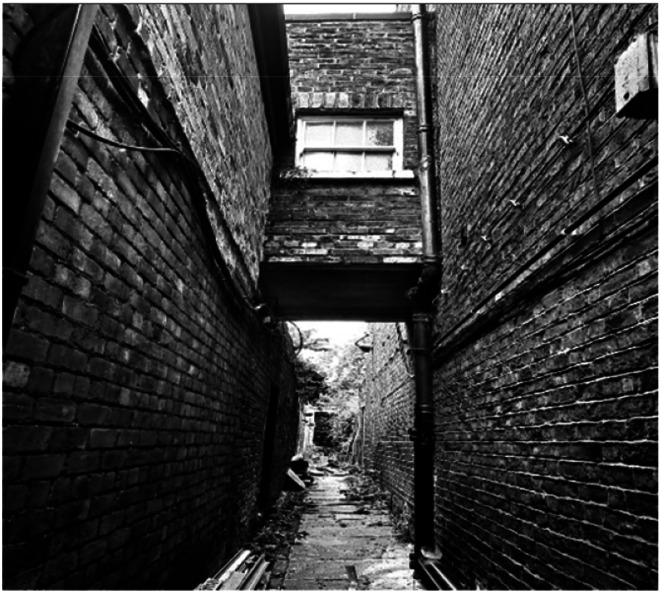
Back-alley photo presented by Selma.

Eighty percent of the women in this study sought medical support to manage symptoms or out of curiosity due to various health and social challenges such as struggling with anger management, that could have been related to menopause. Others like Selma (57, mixed race-Black and African origin) exclaimed that the support offered about menopause is also insufficient. She expresses her experience of seeking medical support saying: ‘*There was no support, doctors didn’t really talk about menopause… I had nobody*’*.* Selma provided a back-alley photo (Figure 4) to signify her experience of seeking support:

She added: ‘*That really just sums it up. It's like it's just a back entry of rubbish and rats…with no ends to it. And that's how I felt…You cannot progress or heal on your own*’.

**Figure 5.**
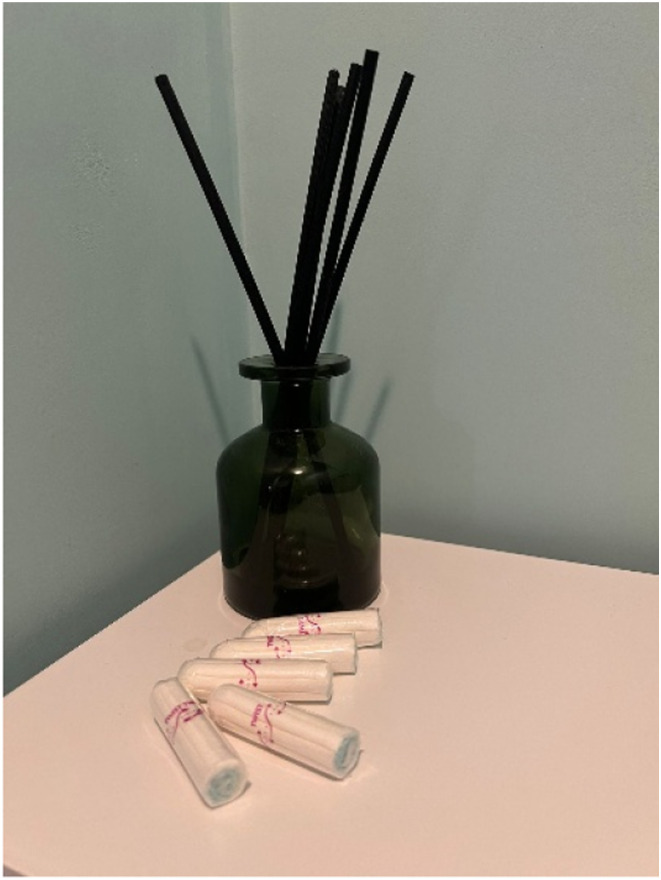
Tampons photo presented by Charlotte.

Owing to the challenges of accessing healthcare, women managed their symptoms in different ways including **self-care.** Charlotte (45, Chinese-Hong Kong heritage), for example, shared a photo of tampons (Figure 5) to signify one of the ways she managed her menopause symptoms:

She said: ‘*I never used tampons in Hong Kong because it wasn’t as common as in the West… But when I came to the United Kingdom, a friend suggested I try tampons because my flow was heavy, and it was messy. She recommended I try it, and once I did, I couldn’t go back… I don't know about other people's menopause symptoms, but for me, menopause made my periods more irregular and heavier…*’

**Figure 6.**
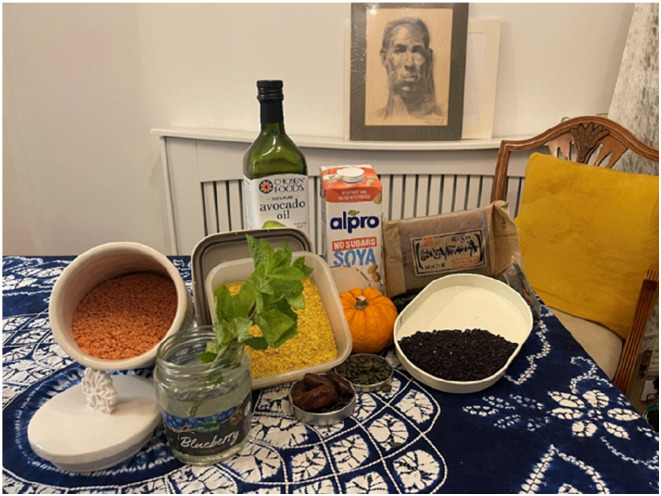
Foods photo presented by Brenda.

**Figure 7.**
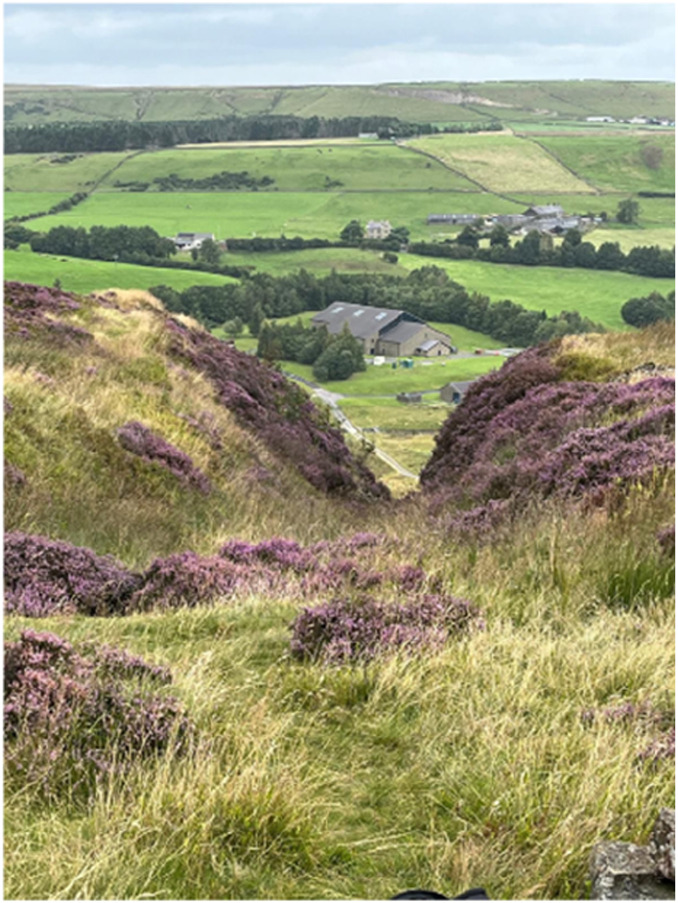
Country-site photo presented by Eva.

**Diet management** was the other method that 80% of the women mentioned as central to managing menopause. Brenda, for instance (53, Chinese-Hong Kong heritage), explains how she became vegan: ‘*…I met two sisters who highly recommend a vegan lifestyle. They told me that I had to change my diet, and it would help me transition through menopause smoothly… They taught me to stop eating meat and dairy, among other things… I also learned how to cook because I didn’t know how to cook before …people say, “You are what you eat,” and it’s true*’*.* Brenda provided a foods photo (Figure 6) to show her diet therapy:

**Exercising** was mentioned by half of the participants to keep the body active both for physical benefits such as bone health and psychological benefits including brain health. Eva (47, Black African origin) provided acountry-site photo (Figure 7) signifying her ventures to exercise by hiking for wellbeing:

She says: ‘*It's sometimes six hours that you're out in the sticks, in the trees, in the air, hugging the sides of a hill*’*.* Adding that she invites a group of Black women to the hikes she says: ‘ *[we] come away feeling that you've had really good company, you know, laughing, being silly, venting* (including about menopause) *… So, you come back completely released*’*.*

The aspect of **community **was strongly recommended by many of the women for the benefits of general human connections that make one feel less isolated, more supported and finding self-worth by being empowered or empowering other women.

### Menopause and the life course

#### Religion and beliefs

**Figure 8.**
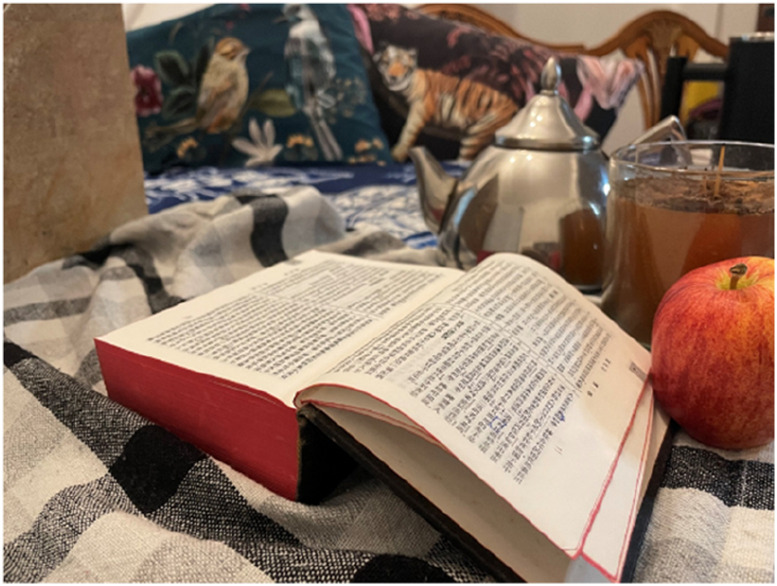
Chinese Bible photo presented by Brenda.

All the women who participated in this photovoice study conveyed that they had certain beliefs or followed a religion (90% were Christians, see Table 1). Discussions of how religion/beliefs affect menopause experiences or vice versa were common especially in line with coping mechanisms where prayers, rituals and teachings were said to help alleviate the impact of symptoms or provide explanations and knowledge of the menopause experiences. Some women said that they prayed for their menopause to come early, this was especially connected to painful periods or inability to bear children. Brenda (53, Chinese-Hong Kong heritage), for example, provided a photo depicting figures (animals and an apple) of the ‘creation story’ with a page opened to the Chinese Bible chapter containing that story (Figure 8):

She said: ‘*[the photo] represents the pain I’ve experienced with my period since I was young. So, for me, menopause is a great joy. Menopause is truly a gift for me, finally, you’ve given it to me! I always longed for my periods to end. I used to suffer so much. Before my period, my emotions would be terrible, irritable and moody… it felt like a big battle. I blamed Eve, saying that because she ate the apple, it was a spiritual release for me’.*

**Table 1.** Participants’ characteristics.

Participant’s characteristics		Number	Percent
**Age range**	45–50	6	60%
	51–55	2	20%
	56–60	2	20%
**Ethnicity**	Black African	3	30%
	Black Caribbean	1	10%
	Mixed White and Black African	2	20%
	Chinese-Hong Kong	2	20%
	Chinese-Mainland	2	20%
**Years lived in the United Kingdom**	0–5 years	3	30%
	6–25 years	3	30%
	26–45	2	20%
	46–60	2	20%
**Religion**	Prefer not to say	1	10%
	Christian	9	90%
**Number of living children**	None	3	30%
	1	1	10%
	2	3	30%
	3	2	20%
	4	1	10%
**Menopause status**	Perimenopause	6	60%
	Post menopause	4	40%

**Figure 9.**
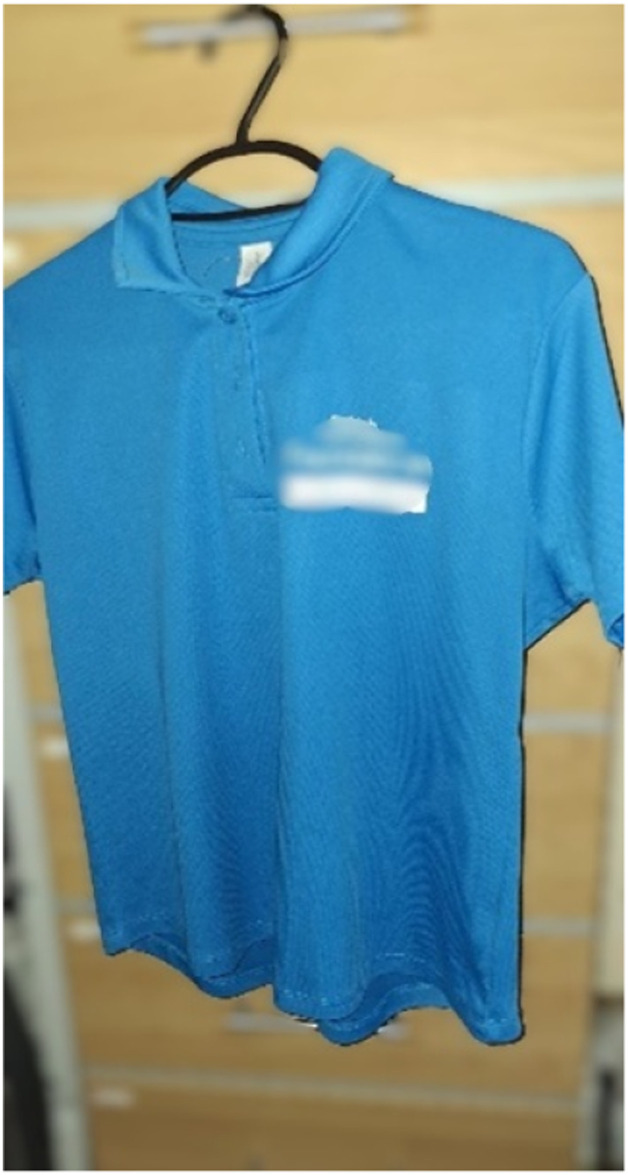
Work uniform photo presented by Louie.

**Work**, as part of the whole life course which can be affected positively or negatively, Louie (58, Chinese-Mainland heritage), expressed her experience in relation to menopause by sharing a photo of her work uniform (Figure 9):

She said: ‘*…it’s very meaningful to me. The company asked me to do some training to teach elderly people exercise… I became an instructor, and it helped me too because I had to learn it first before teaching them. When doing exercise, the whole body’s circulation improves, and your hands and feet don’t feel so cold… my body might be starting to age because of menopause, but doing exercise could slow down the process*’

The menopause transition period was depicted by many women as time of seeking new directions in terms of vocation, some of which is connected to the prospective of empty nest status for those with children, and less or more grounded but calmer ventures for women without children who desired to contribute more to the communities.

**Figure 10.**
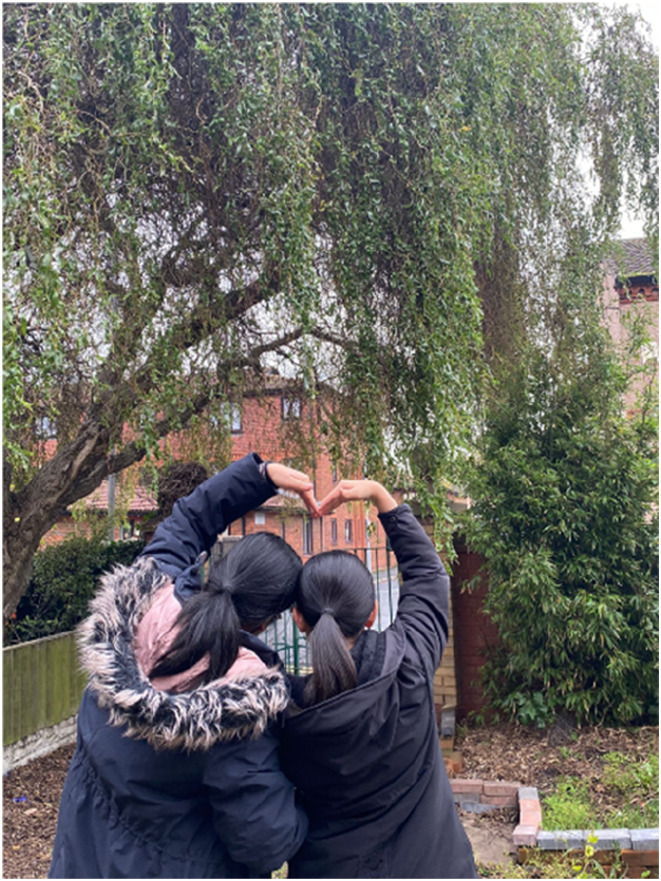
Photo of participant and her daughter, presented by Jane.

**Family** was discussed as a source of support and inspiration by most of the women during the menopause transition, however, conflicts arising between the women with their children or with elderly parents were a common theme. For example, Jane (47, Chinese-Mainland heritage) who is one of the seven women who had between one and four children, provided a photo (Figure 10) of her and one of her daughters highlighting the tension she had with her oldest daughter that affected the other children:

She said: ‘*Before, my relationship with my oldest daughter was really bad…I told her that I was going through menopause’. She would always say, ‘Do you know it’s the hormonal changes of puberty that make me so irritable?*’ *Actually, she was right. Later, I told her, ‘I’m also going through hormonal changes due to menopause, and I get irritable too…. once we talked it through, everything started to get better… I now try to get the support of my family*’*.*

**Ageing** was also a common theme discussed in this study with some women providing visuals relating to body changes including changes on skin showing blackheads and other pigmentations that tend to affect elderly skin. One woman provided a photo of a tree during autumn season with some leaves fallen and some still on the branches. She expressed how she felt her body was responding to the changes connected to ageing. Others discussed about death, for instance, one woman provided a photo of two roses, one with natural white colour and another rose with died blue colour, both of which were in one photo crisscrossing at the stalks that signified the good and bad experiences intertwined.

## Discussion

Women from Black and Chinese heritage communities in the Northwest of England were invited to tell their stories with the aid of photographs (photovoice research method) that represented their menopause experiences. Ten women agreed to participate. Their contribution prompted discussion and reflection that revealed insights not only on their experiences but also their perspectives and emotions, which would not have otherwise been captured using traditional research methods. Photovoice offers unique insights that encourage participants to open up about personal stories and researchers to think outside the box as they analyse visual data.^8^ Evidence has shown that hidden aspects of human experiences can be uncovered using such creative methods and findings can evoke a greater understanding and empathy.^9^ This paper has presented findings incorporating photos shared by the participants about their menopause experiences and the inference thereof.

As research has previously suggested certain ethnic communities are less likely to interact with healthcare practitioners,^3,10^ this study has shown that conversely, women feel that their doctors do not talk about menopause or initiate discussions with their patients. This could help explain why there is less interaction from women of minority ethnic communities with their healthcare providers as they expect the conversation to be initiated by their doctors.

Women in this study who experienced debilitating symptoms related to menopause expressed their struggles which often led to isolation. Some sought support based on their symptoms, but many feared that consultations with healthcare providers would result in prescription of medication. These responses could be attributed to very little being known about women from minority ethnic groups,^3^ thus leading to interventions that are incompatible with the women’s biological, social or cultural concepts.

Self-care, diet management and exercise were therefore the ultimate menopause management strategies for most women in this study. However, some needed to adapt their sanitary self-care sometimes using means that are not common in their culture. Many women also had to discover new diet options and eating habits, most of which encouraged vegetables or plant-based foods. Extra effort was required to engage in exercises that improved health conditions such as strengthening bones and flexing joints. Such endeavours have proven to improve menopausal symptoms^11^ with less active, sedentary lifestyles being highlighted as a causal factor for more severe symptoms at menopause.^12^ This may be one of the reasons why many women from minority groups do not end up in mainstream healthcare, and as a result, less is known about their experiences. These management strategies may also shape their expectations of healthcare advice.

Menopause management strategies were communicated and experienced in community groups; however, as described earlier by some of the participants, isolation is common especially in women with debilitating symptoms. All the women commended community as an absolute necessity during the menopause transition, indicating that they appreciated sharing of challenges and solutions, knowledge and experiences leading to healing and empowerment. The collectivist culture with an emphasis on social cooperation and decision making^13^ requires consideration when discussing menopause with ethnic minority women.

The focus on life course in this study revealed women’s menopause experiences being impacted by religion, work, family and ageing, or vice versa, where their previous understanding and life objectives were challenged, sometimes requiring change. This study has shown that influence of these aspects contributed to better coping or exacerbation of symptoms because they determined how the women responded and their perspectives towards menopause and its management. Discussing menopause and not including these aspects poses a narrow focus that ignores the intersectionality model recommended for holistic social, health and wellbeing.^14^

## Strengths and limitations

One strength of this study is that we included more than one ethnic minority groups, which provides a broader perspective. The use of photovoice methodology is another strength that enhanced engagement and empowered participants to express themselves using photos thus providing insights of menopause experiences that would otherwise not have been captured. Limitations are that there are other ethnic minority women who could have further enriched the findings of this study if they were included. Therefore, although the concepts discussed in this study could be reflected in the experiences of minority ethnic women who are not Black or from Chinese heritage, we acknowledge the heterogeneity present between and within the groups.

## Conclusion

The menopause experiences of Black and Chinese heritage women highlighted in this photovoice study show that there are biological, social and cultural aspects reflected in symptoms, self-care, diet, exercise and community that require consideration in any discussion or intervention related to menopause. A life course approach is important because other life events and aspects of religion/beliefs, work, family and ageing affect perspectives about menopause and how one responds to it including its management. There is a particular emphasis on an intersectionality approach to ensure that presented queries and issues are not considered or handled in isolation.

## Data Availability

Data that support the findings of this study are available on request from the corresponding author SW or the Principal Investigator SP. The data are not publicly available because they contain information that could compromise the privacy of the participants of this study.*
